# Editorial: Metagenomics for epidemiological surveillance in One Health

**DOI:** 10.3389/fmicb.2023.1191946

**Published:** 2023-03-31

**Authors:** Michael E. von Fricken, Mel C. Melendrez, Yvonne-Marie Linton, Ratree Takhampunya

**Affiliations:** ^1^Department of Global and Community Health, College of Public Health, George Mason University, Fairfax, VA, United States; ^2^Department of Biology, Anoka-Ramsey Community College, Coon Rapids, MN, United States; ^3^Walter Reed Biosystematics Unit, Smithsonian Museum Support Center, Suitland, MD, United States; ^4^Department of Entomology, Smithsonian Institution – National Museum of Natural History (NMNH), Washington, DC, United States; ^5^Walter Reed Army Institute of Research, One Health Branch, Silver Spring, MD, United States; ^6^Department of Entomology, United States Army Medical Directorate—Armed Forces Research Institute of Medical Sciences (AFRIMS), Bangkok, Thailand

**Keywords:** metagenomics, biosurveillance, One Health, next generation sequencing (NGS), vector-borne diseases

The highest burden of endemic, neglected, and emerging zoonotic diseases occur in low and middle-income countries (LMICs) (Worsley-Tonks et al., 2022). However, limited resources, surveillance infrastructure and access to advanced molecular techniques in these regions can severely impact the rapid detection and characterization necessary to mitigate and respond to outbreak events. Metagenomics approaches continue to become less expensive, and now represent a potentially cost-effective approach to conduct biosurveillance and monitor molecular epidemiology (Ko et al., 2022). Compared to PCR, which only detects pathogen based on specific primers, metagenomic agnostic sequencing allows for the identification of known and unknown microbes (viruses, bacteria and protozoans) from a single sample (Govender et al., 2021). Forward-facing portable sequencing platforms such as the MinION can provide expansive knowledge of circulating pathogens from a wide array of field-collected samples, including arthropod vectors, soil and water samples and a variety of swabs and bloods from vertebrates, both in the field and in basic laboratory conditions (Gardy and Loman, 2018; Achee et al.). Using metagenomics approaches, detection of known, emergent, and emerging zoonotic pathogens of human and veterinary importance can be made available to decision-makers, researchers, and policymakers in near real-time, to inform effective containment or mitigation efforts to minimize public health impacts.

The nine publications presented here in this Research Topic capture a wide range of settings that metagenomics can be applied to through a One Health lens, including outbreak response, clinical management, biosurveillance, and disease monitoring within the human animal interface. Publication summaries can be found in Table 1.

**Table 1:** Summary of special edition publications as they relate to metagenomics, surveillance, and One Health.

**Article title**	**Key findings**
Dissemination of resistant *Escherichia coli* among wild birds, rodents, flies, and calves on dairy farms	Animal, arthropod, and environmental samples were collected on 57 Swedish dairy farms. Isolates from different sources on the same farm generally clustered together, with resistant *E. coli* detected from calves and scavenger animals with little genomic differences, suggesting interspecies transfer of pathogens. Transmission of *E. coli* between species highlights the potential risk of AMR spread in areas with low antimicrobial use.
Metagenomic next-generation sequencing reveals the profile of viral infections in kidney transplant recipients during the COVID-19 pandemic	NGS was utilized in kidney transplant recipients for the detection of viral infections during the COVID-19 pandemic. In 48/50 clinical samples, 15 types of viruses were detected, including cytomegalovirus, Torque teno virus, human alpha and beta herpesviruses, JC, BK, and WU polyomaviruses, primate bocaparvovirus 1, simian virus 12, and volepox virus.
A rapid, whole genome sequencing assay for detection and characterization of novel coronavirus (SARS-CoV-2) clinical specimens using nanopore sequencing	A modified SARS-CoV-2 nanopore sequencing assay was developed based on the ARTIC protocol for secluded, low resource settings. When analyzing six of these sequences for mutations, there were existing and unique changes in the sequence, with three occurring in the Spike protein. Rapid characterization of SARS-CoV-2 using this data may be informative for subsequent targeted control measures.
Metagenomic investigation of ticks from kenyan wildlife reveals diverse microbial pathogens and new country pathogen records	Detected a variety of microbial species in ticks collected from the environment, wildlife, and domestic animals, using NGS. A major advantage of agnostic pathogen identification techniques resulted in the detection of several emerging zoonotic agents and resulted in the first ever detection of Jingmen tick virus in Kenya.
Metagenomic profiles of dermacentor tick pathogens from across mongolia, using next generation sequencing	Dermacentor ticks (*n* = 1,773) from 15 provinces of Mongolia were screened using NGS. *Rickettsia* spp. was detected in 88.33% of screened tick pools. *Anaplasma* spp. and *Bartonella* spp. were detected in 3.18% and 0.79% of pools, respectively. *Anaplasma* spp. was only detected in ticks collected from livestock and this the first report of *B. melophagi* in ticks from Mongolia.
Genomic and virologic characterization of samples from a shipboard outbreak of COVID-19 reveals distinct variants within limited temporospatial parameters	Using whole-genome sequencing on COVID-19 positive swabs, produced 18 viral genomes, of which, seven were unique variants. High rates of vaccination may limit viral evolution therefore reducing the spread of COVID-19. During outbreaks, sequencing surveillance can prove vital toward understanding viral molecular epidemiology.
The remote emerging disease intelligence—NETwork	The REDI-NET consortium was established to improve pathogen surveillance in the United States, Kenya, and Belize utilizing a One Health approach. Metagenomic screening pathways are described in detailed frameworks between tiered laboratory systems, where analysis pipelines will allow for the formation of disease dashboards, risk maps, and models.
Discovery of *Rickettsia* spp. in mosquitoes collected in georgia by metagenomics analysis and molecular characterization	Study used NGS to examine mosquitoes as potential vectors of *Rickettsia* spp. pathogens. Some 475 pools of *Aedes, Culex*, and *Culiseta* mosquitoes collected in Georgia from 2018 to 2019 were screened, 33 of which tested positive for rickettsial DNA. Genus-specific qPCR and multi-locus sequence typing identified a *Rickettsia* spp. closely related to *R. bellii*.
Diagnostic Efficiency of metagenomic next-generation sequencing for suspected spinal tuberculosis in china: A multicenter prospective study	NGS was used to detect pathogens in patients with suspected spinal tuberculosis (TB). A 100 patients were enrolled and pathogens were found in 82 patients. Patient findings included TB (*n* = 37) and 45 patients infected with other bacteria. The sensitivity of the NGS assay in patients with a spinal infection was higher than that of culture and pathological examination, but not statistically different than Xpert and T-SPOT.TB.

## Metagenomics during outbreaks

During an outbreak investigation, Cer et al., showed the utilization of metagenomics by comparing genomic variants to measure human–human spread and mutation rates of the COVID-19 virus. Comparing two different outbreaks of COVID-19—one in a group prior to vaccine development and one in a group with high vaccination rates—a larger number of variants were observed within the unvaccinated group. The study highlighted further questions and discussion on the impacts of sampling bias, the impact of purifying selection in creating clonal viral populations in vaccinated individuals, and how that can inform and support vaccination efforts.

Pandemics such as the Ebola in 2014–2016 and COVID-19 have shown that global security is compromised when response requires advanced training, special tools, and established infrastructure to accurately survey and assess the outbreak situation, where delays ultimately cost lives. It was during the Ebola outbreak that portable sequencing technology (MinION) was piloted in the field to speed up detection and public health response with a simpler, pared down protocol and analysis workflow. COVID-19 sequencing protocol using the ARTIC protocol from Illumina can be time consuming, limiting the potential use of molecular epidemiology for control measures. To reduce sequencing time during an outbreak, Arévalo et al., modified a COVID-19 whole genome sequencing assay that produced high quality metagenomics data relatively quickly in an isolated, low resource setting. Adaptation of current protocols to LMIC infrastructure and resources while providing a quick turnaround for results will be invaluable in controlling future outbreaks.

## Clinical applications of NGS

Clinically, metagenomics can be used to identify unknown or rare pathogens that might go undetected using traditional methods. An example of this was outlined by Tian et al., to detect viral infections in kidney transplant recipients (KTRs) during the COVID-19 pandemic. An NGS approach detected rare viruses in multiple sample types including bronchoalveolar lavage fluid (BALF), urine and blood. This method identified infections or co-infections in vulnerable immunosuppressed kidney transplant recipients, which was then used to inform treatment strategies.

Li et al., used NGS on clinical samples of patients who were suspected of having spinal tuberculosis (TB). Over half of the samples (45/82) tested had bacteria that was not *Mycobacterium tuberculosis*. This approach quickly and simultaneously confirmed patients with spinal infection if they had TB or non-TB infections. This approach also distinguished and identified other pathogens in these patients, which helped guide patient's clinical treatment. This study evaluated the efficacy and application of NGS as clinical diagnostic assay for suspected spinal TB infection in clinical laboratories.

## Biosurveillance

Altantogtokh et al., tested pools of *Dermacentor nuttalli* ticks (*n* = 1,773) from 15 provinces of Mongolia using NGS. *Rickettsia* spp. was detected in 88.33% of screened tick pools, with Khentii aimag having the highest detection rate for *Rickettsia* spp. *Rickettsia raoultii* and *Rickettsia sibirica* were detected within this study, aligning with previous findings that applied NGS to livestock samples in Mongolia (Chaorattanakawee et al., 2022). *Anaplasma* spp. and *Bartonella* spp. were detected in 3.18 and 0.79% of pools, respectively, with *Anaplasma* spp. only detected in ticks collected from livestock, as seen elsewhere (von Fricken et al., 2020). This study represents the first detection of *Bartonella melophagi* in ticks tested from Mongolia.

Pollio et al., discovered a novel *Rickettsia* spp. in mosquitoes which shared a close identity with *Rickettsia bellii* based on data from NGS. While it is unknown if *R. bellii* is pathogenic in humans, detecting this microorganism in mosquitoes may hold value down the road should this disease emerge. The use of metagenomics to screen mosquito samples elsewhere in the world may hold value for the identification and characterization of potential pathogenic *Rickettsia* spp. within an atypical vector host.

## Metagenomics at the human-animal interface

Human-livestock transmission is one mode for infection of zoonotic pathogens. The likelihood of infection is increased as animals pass pathogens between species similar to the spread of *Escherichia coli* in Hickman et al., This interspecies exchange is conducive to pathogen mutations, and passage of mobile genetic elements or plasmids *via* clonal or horizontal gene transfer, increasing the risk of spreading to humans potentially increasing the severity of infection, and resistance to antibiotic interventions.

Ergunay et al., used NGS to screen 75 blood-fed ticks (*Rhipicephalus* spp. and *Amblyomma* spp.) collected from wild and domestic animals in Kenya for pathogens. Fifty-six human or veterinary pathogenic bacterial species were detected including *Escherichia coli* (62.8%), *Proteus mirabilis* (48.5%), *Coxiella burnetii* (45.7%), and *Francisella tularensis* (14.2%). Additional pathogens detected include fungal species, filarial pathogens, protozoa, and environmental and water/foodborne pathogens. Jingmen tick virus was detected within 13% of these samples, representing the first report of this virus in Kenya. Interestingly, in these wild and domestic animal blood-bags, human pathogens, including *Plasmodium falciparum* were detected, indicating back spill-over of pathogens of human pathogens to animals.

Finally, the adoption of a One Health focused surveillance strategy is described in detail by Achee et al., through the Remote Emerging Disease Intelligence—NETwork (REDI-NET), which has implemented MinION-based xenosurveillance strategies in the United States, Kenya and Belize. Comprehensive standard operating procedures (SOPs) were developed for the deployment of metagenomic surveillance at high-risk disease envelopes. The ultimate goal of REDI-NET is to apply agnostic pathogen detection to a variety of sample types including water, ticks, soil, and leeches across Gold- (reach-back laboratories with greater infrastructure) and Silver- (remote, field-forward laboratories) tiered laboratory systems. This study outlined the framework required for working with metagenomic data, efficient data pipeline strategies, and systematic processes for collection and testing of samples for deeper analysis.

This Research Topic highlights the diversity of metagenomic application across disciplines, geographical regions, and both research and clinical settings. The field of metagenomics is growing rapidly, with all papers in this Research Topic published within the past year. Utilizing metagenomics from a One Health perspective will expand surveillance and characterization of existing and new pathogens in humans and animals, which will likely continue to be adopted into existing systems as next generation sequencing becomes more accessible and cost-effective.

## Author contributions

All authors listed have made a substantial, direct, and intellectual contribution to the work and approved it for publication.
